# Cyclic di-AMP alleviates periodontitis by activating PI3K/Akt/Nrf2 pathways

**DOI:** 10.3389/fcimb.2025.1560155

**Published:** 2025-03-14

**Authors:** Kaihua Luo, Qinrui Wu, Zhengyi Li, Yajie Wu, Zhifei Su, Fangjie Zhou, Qinyang Li, Biao Ren, Yuqing Li, Jiyao Li, Xian Peng

**Affiliations:** ^1^ State Key Laboratory of Oral Diseases & National Center for Stomatology & National Clinical Research Center for Oral Diseases, West China Hospital of Stomatology, Sichuan University, Chengdu, Sichuan, China; ^2^ Department of Cariology and Endodontics, West China Hospital of Stomatology, Sichuan University, Chengdu, Sichuan, China

**Keywords:** c-di-AMP, PI3K/Akt signaling pathway, second-messenger systems, periodontitis, host-pathogen interactions, microbial dysbiosis

## Abstract

Emerging research demonstrates the regulatory effects of c-di-AMP, a bacterial-derived small molecule secondary messenger, on host immune responses and promoting resistance against infection-related diseases. This study aims to elucidate the role of c-di-AMP in the occurrence and development of periodontitis. Using model of ligation-induced periodontitis, we observed that c-di-AMP effectively alleviated alveolar bone resorption. Transcriptomic sequencing in mice gingival tissues demonstrated that treatment with c-di-AMP led to a significant upregulation of the PI3K/Akt signaling pathway and its key components, including Akt3. Concurrently, we observed an upregulation of the cGMP/PKG signaling pathway. To validate our findings, we treated gingival epithelial cells with c-di-AMP and confirmed the activation of the PI3K/Akt pathway by c-di-AMP in gingival epithelial cells. Under LPS-induced inflammation, c-di-AMP significantly suppressed the release of inflammatory factors (such as IL-6 and TNF-α) from gingival epithelial cells. Moreover, key components of the PI3K/Akt pathway, including Akt, and downstream inflammation regulatory gene Nrf2, were upregulated, which were also confirmed at the protein level. Collectively, this study demonstrates that c-di-AMP definitely plays a role in alleviating periodontitis. Our findings highlight the mechanisms by which c-di-AMP modulates periodontitis, including activating the PI3K/Akt pathway and potentially involving the cGMP/PKG pathway, ultimately contributing to improved immune defense and maintenance of bone homeostasis.

## 1 Introduction

Periodontitis is a chronic inflammatory disease affecting the tissues surrounding and supporting the teeth, characterized by progressive destruction of these structures (Kinane et al., 2017). The pathogenesis is initiated by the accumulation of bacteria in the dental plaque, disrupting oral microbial homeostasis and enabling overgrowth of periodontal opportunists such as *Porphyromonas gingivalis* (Gasmi Benahmed et al., 2022). This perturbs host-microbe interactions, triggering inflammatory responses and progressive destruction of periodontal tissues (Lamont et al., 2018).

Localized inflammation elicits activation of resident cells (epithelial cells, fibroblasts, endothelial cells, and macrophages), recruitment of inflammatory cell types (monocytes, lymphocytes, and neutrophils), and subsequent release of inflammatory mediators including reactive oxygen species (ROS) from neutrophils and cytokines (interleukin IL-1β, IL-6, IL-8, and tumor necrosis factor alpha) that drive periodontal tissue damage (Hajishengallis, 2015, 2020). While physiological ROS levels mediate antimicrobial defense and immune regulation (Sczepanik et al., 2020), excessive ROS can damage cellular proteins and DNA, disrupting cell cycle progression and growth (Kanzaki et al., 2017).

The PI3K/Akt signaling pathway plays a pivotal role in regulating inflammatory responses and innate immune cell activation. One of the primary mechanisms through which PI3K/Akt modulates inflammation is by recruiting and activating innate immune cells, including macrophages and neutrophils (Acosta-Martinez and Cabail, 2022; Vergadi et al., 2017; Zhou et al., 2018). Akt is a serine/threonine kinase and it participates in the key role of the PI3K signaling pathway. Upon activation by survival factors, Akt can suppress apoptosis by phosphorylation and inactivation of key regulatory components. In the mammalian genome, despite their high similarity of 80%, three Akt genes exist (Akt1, Akt2, and Akt3) exert non-redundant, and display varying expression at both the mRNA and protein levels and serve unique roles in cell function. The isoforms Akt1 and Akt2 are involved in apoptosis and insulin signaling (Hinz and Jücker, 2019). Emerging evidence suggests that Akt3 influences inflammatory responses by maintaining central nervous system integrity during neuroinflammation (DuBois et al., 2019), and modulating endothelial autophagy, inflammation, apoptosis and angiogenesis (Tang et al., 2021). As a key regulator of endothelial autophagy, Akt3 plays a crucial role in protecting vascular homeostasis under inflammatory conditions. Restoration of Akt signaling has been linked to enhanced autophagy, reduced oxidative stress, and suppression of inflammatory markers, thereby preventing endothelial injury (Zhang et al., 2021). Furthermore, Akt3 influences pro- and anti-inflammatory cytokine production. In experimental models of inflammatory kidney disease, Akt3 modulation reduced the secretion of TNF-α, IL-6, and IL-1β, while enhancing anti-inflammatory cytokine responses (Chen et al., 2024).

The activation of the PI3K/Akt pathway induced by early mild infections can promote the survival of gingival epithelial cells and downregulate the inflammatory response (Yilmaz et al., 2004). Downstream of PI3K/Akt, the transcription factor nuclear factor erythroid 2-related factor 2 (Nrf2) upregulates antioxidant and anti-inflammatory genes (Xi et al., 2021). Akt3 reportedly regulates Nrf2 activity and reactive oxygen species (ROS) levels (Zhao et al., 2017). In severe periodontitis characterized by extensive clinical attachment loss and bone destruction, sustained local ROS and declining antioxidant capacity associate with Nrf2 downregulation in neutrophils. And the excessive and persistent ROS coupled with impaired plasma antioxidant mediates oxidative damage within periodontal tissues, contributing to intractable inflammation (Chiu et al., 2017). Nrf2’s protective effects help maintain periodontal health despite constant bacterial and immune interactions (Hasturk et al., 2012; Tian et al., 2022).

Cyclic di-AMP (c-di-AMP) is a novel bacterial second messenger discovered in bacteria and archaea (Fahmi et al., 2017). This widely produced bacterial product not only involves various physiological process in of bacteria, such as metabolism, cell osmotic pressure maintenance, response to DNA damage, formation of biological membranes, but can also be recognized by host cells to trigger host immune responses (Cheng et al., 2022; Witte et al., 2013). Functioning as a novel pathogen-associated molecular pattern (PAMP), c-di-AMP elicits recognition by pattern recognition receptors (PRRs) within the host’s immune system (Devaux et al., 2018). The STING pathway is considered to be the classical pathway through which c-di-AMP influences the immune response. Activation of STING leads to the induction of type I interferon (IFN) expression during bacterial infections, a response that is thought to underlie the enhancement of acquired immunity by c-di-AMP (Rueckert et al., 2017; Woodward et al., 2010). Recent investigations suggests that bacterial c-di-AMP and STING activation may also contribute to the pathogenesis of periodontal disease. In this context, STING activation triggered by oral bacteria can modulate inflammation and immune responses in periodontal tissues (Elmanfi et al., 2021). Moreover, STING activation may also engage in crosstalk with PI3K/Akt signaling, affecting cytokine production and cell metabolism (Ahn and Barber, 2019; Liu et al., 2022). C-di-AMP is widely distributed among oral bacteria and within biofilms, where it plays a critical role in modulating bacterial growth, metabolism, colonization, biofilm formation, and cell wall homeostasis (Fahmi et al., 2017; Kim et al., 2015; Witte et al., 2013). Our previous work in *Streptococcus mutans* showed that reduced intracellular c-di-AMP levels correlated with impaired biofilm formation and, consequently, diminished cariogenic virulence (Peng et al., 2017). Similarly, in *Porphyromonas gingivalis*, a key periodontal pathogen, a well-developed system for both c-di-AMP synthesis and hydrolysis has been identified, suggesting that this second messenger may influence the regulation of periodontal infections (Peng et al., 2016).

In this study, we discovered that c-di-AMP can have a protective effect against periodontitis independent of STING signaling. Instead, c-di-AMP activates the PI3K/Akt/Nrf2 pathway to suppress inflammation, leading to reduced tissue damage and bone loss. Specifically, we found that c-di-AMP treatment upregulates PI3K and Akt expression and enhances Akt phosphorylation in gingival tissues and cells. The activation of PI3K/Akt signaling likely induces Nrf2-mediated antioxidant and cytoprotective responses that mitigate periodontal inflammation and bone resorption.

## 2 Materials and methods

### 2.1 Reagents

The c-di-AMP (purity ≥ 98%) was obtained from Sigma-Aldrich technology, Co. (Shanghai, China) and diluted in PBS for *in vivo* and *in vitro* experiments. LPS from *Porphyromonas gingivalis* was obtained from Sigma-Aldrich technology, Co. (Shanghai, China). The Akt inhibitor MK-2206 and the STING inhibitor C176 were purchased from APExBIO (USA). Stock solutions of MK-2206 (10 mM) and C176 (10 mM) were prepared in dimethyl sulfoxide (DMSO) and further diluted in PBS to achieve final working concentrations of 1 µM for MK-2206 and 1 µM for C176 in experimental conditions.

### 2.2 Animal experiments

Six-week-old male healthy C57BL/6 mice were purchased from Chengdu Dossy Experimental Animals Co., Ltd. (Chengdu, China) and fed in a temperature-controlled room, with a 12 h light-dark cycles. The study was conducted according to the guidelines of ARRIVE and the experimental protocols were approved by the Animal Care and Use Committee of West China Hospital of Stomatology Hospital, Sichuan University, Chengdu China (WCHSIRB-D-2020-031).

**Figure 1:**
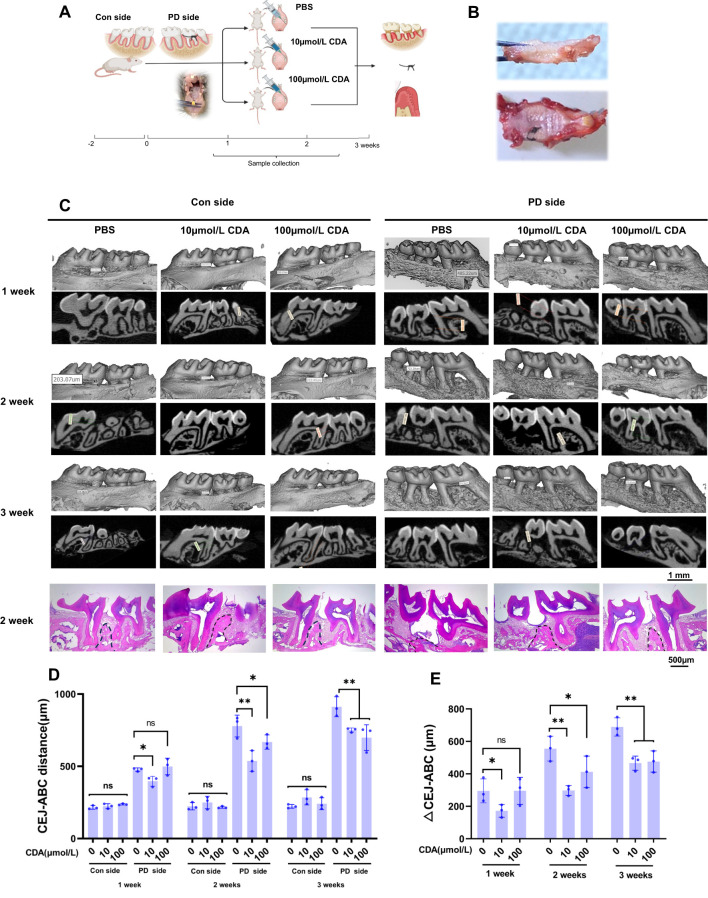
C-di-AMP inhibits bone destruction in ligation-induced periodontitis. **(A)** Establishment of mice periodontitis model, grouping, and schedule of CDA treatment in schematic illustration. Sample size: n = 9 per group, with 3 mice assessed at each designated time point. Sample collection was implemented in 1 week, 2 weeks and 3 weeks including alveolar bone, ligature with microbe and gingiva. **(B)** Alveolar bone with ligature attached on the second molar. Removing the ligature, we can observe attachment loss and a decrease in alveolar bone height on the second molar. **(C)** Three-dimensional or two-dimensional representative micro–computed tomography images of maxillae in 3 groups. Both control side and periodontitis side were represented at 3 weeks. The last row shows the H&E staining results of the maxillary bone at the 2-week time point for each group. The position of the alveolar crest is marked with a black dashed line. **(D)** The distance from the cemento-enamel junction (CEJ) to the alveolar bone crest (ABC) was measured to quantitatively characterize alveolar bone absorption. **(E)** And the difference value of PD side and Con side, ΔCEJ-ABC was served as an indicator of the severity of alveolar bone resorption. *p < 0.05, **p < 0.01.

After a 2-week acclimatization period, mice were randomly divided into three groups (n = 9 per group, with 3 mice assessed at each designated time point): control, 10 μM c-di-AMP and 100 μM c-di-AMP intervention. The concentrations of 10 μM and 100 μM were selected based on preliminary pilot studies and our previous work (Wu et al., 2023). Mice were first anesthetized with isoflurane (5% for induction and 2% for maintenance) delivered via an anesthesia chamber, a method widely used in rodent studies due to its rapid onset and precise control of anesthesia depth. A ligation-induced periodontitis model was then established on the left side, with the control group receiving 0.9% NaCl solution and the intervention groups receiving either 10 μM or 100 μM c-di-AMP solution. After model establishment, 25 μL of the respective solution was locally injected at six sites around the first maxillary molar. Ligatures were checked every two days. The experiments were started with mice at 8-week of age after 1-week of adaptive feeding and terminated at the 11th week (**Figure 1A**). The study was conducted in two independent experimental rounds to ensure the reproducibility of the results.

All mice were sacrificed humanely using established protocols to ensure they were anesthetized and unconscious before sample collection. Euthanasia was performed using an overdose of pentobarbital sodium. Mice were administered an intraperitoneal injection of pentobarbital sodium at a dose of 150 mg/kg body weight. This dosage ensured rapid induction of deep anesthesia followed by euthanasia. Death was confirmed by the absence of heartbeat and respiration, and cervical dislocation was performed as a secondary method to ensure death.

Alveolar bones and gingival tissue were harvested from the mice, postfixed with 4% paraformaldehyde overnight, and stored in 70% ethanol at 4°C for long-term storage. Silk samples were carefully collected before euthanasia in sterile Eppendorf tubes and kept at -80℃ for future analysis. Alveolar bone tissue was collected from all mice for μCT analysis (Miyajima et al., 2014; Morelli et al., 2018; Rekhi et al., 2021). And only 2-week alveolar bone with gingival tissue were collected for H&E staining (Leite et al., 2017; Marchesan et al., 2018).

### 2.3 Micro-computed tomography analysis

The microarchitectural properties of alveolar bone tissues were imported with µCT system. Parameter settings were as follows: the scanning was performed at 70 kV and 200 μA with 300 ms integration time at a voxel resolution of 10 μm. The images were reconstructed using mimics (version 21.0, Materialise N.V., Leuven, Belgium). The results of BV/TV, BMD, and CEJ-ABC were obtained within the region of interest, which surrounds the second molar. CEJ-ABC was determined by measuring the average distance between the alveolar bone crest (ABC) and the cementoenamel junction (CEJ) in the sagittal images. And ΔCEJ-ABC refers to the difference between CEJ-ABC on the left side and CEJ-ABC on the right side. Trabecular bone volume fraction (Bone volume/Total volume, BV/TV) was used to analyze the degree of trabecular bone resorption.

### 2.4 Hematoxylin and eosin staining

To evaluate the severity of the periodontitis condition and the height of the alveolar crest, Hematoxylin and Eosin (H&E) Staining was performed. Maxillary bone samples were fixed in 4% paraformaldehyde at 4°C for 24 h, decalcified in 10% EDTA (pH 7.4) for 4 weeks, embedded in paraffin, and sectioned into 10-μm sagittal slices. Sections were deparaffinized, rehydrated, stained with hematoxylin (5 min) and eosin (30 sec), dehydrated, cleared in xylene, and mounted. Alveolar bone resorption was assessed by identifying the alveolar bone crest (ABC) position on H&E-stained sections, with a black dashed line marking its approximate height.

### 2.5 16S rRNA sequencing and analysis

The microbiomes of silk ligatures were analyzed using 16S ribosomal RNA (rRNA) sequencing. Total genomic DNA was extracted using the QIAamp DNA Mini Kit (QIAGEN) following the manufacturer’s instructions. DNA concentration and purity were assessed using a NanoDrop 2000 (Thermo Fisher Scientific), ensuring an average yield of ≥20 ng/µL, and integrity was verified by agarose gel electrophoresis. The genome DNA was used as template for PCR amplification with the barcoded primers and Tks Gflex DNA Polymerase (Takara). For bacterial diversity analysis, V3-V4 variable regions of 16S rRNA genes was amplified with universal primers 343F and 798R performed by oebiotech Technology Co., Ltd, Shanghai, China.

The microbiomes of silk ligatures were analyzed using 16S ribosomal RNA (rRNA) sequencing. Total genomic DNA was extracted using the QIAamp DNA Mini Kit (QIAGEN) following the manufacturer’s instructions. DNA concentration and purity were assessed using a NanoDrop spectrophotometer (Thermo Fisher Scientific), ensuring an average yield of ≥20 ng/µL, and integrity was verified by agarose gel electrophoresis. Purified DNA was used as a template for PCR amplification of the V3-V4 hypervariable regions of the 16S rRNA gene using barcoded universal primers 343F and 798R and Tks Gflex DNA Polymerase (Takara, Japan).

### 2.6 16S rRNA sequencing and analysis

Total RNA was extracted from gingival tissue and human gingival epithelial cells using the TRIzol reagent (Invitrogen, USA) following the manufacturer’s protocol. RNA purity and concentration were assessed using a NanoDrop spectrophotometer (Thermo Fisher Scientific, USA), and RNA integrity was evaluated with an Agilent 2100 Bioanalyzer (Agilent Technologies, USA) to ensure an RNA integrity number (RIN) ≥ 7.0. For library construction, mRNA was enriched from total RNA using oligo (dT) magnetic beads, followed by fragmentation, cDNA synthesis, and adapter ligation using the NEBNext Ultra RNA Library Prep Kit (New England Biolabs, USA). The purified libraries were PCR-amplified, and final sequencing libraries were quantified using a Qubit Fluorometer (Thermo Fisher Scientific, USA) and validated with an Agilent 2100 Bioanalyzer.

### 2.7 Cell cultures

Human gingival epithelial cells (HGECs, 3rd passage) were obtained from Warner Biotechnology Co., Ltd. HGECs were cultured and seeded at a density of 1 × 10^5^/mL in a growth medium (DMEM) in 6-well plates overnight. Different doses of c-di-AMP (0, 2, 6 μM) were added to the HGECs, and the samples were incubated for 24h.

### 2.8 qRT-PCR

To validate gene expression, qRT-PCR was performed using the SYBR Green PCR Master Mix (Applied Biosystems, USA) on a QuantStudio 5 Real-Time PCR System (Thermo Fisher Scientific, USA). And cDNA was synthesized using RevertAid First Strand cDNA Synthesis Kit (Thermo Fisher Scientific, USA) following the manufacturer’s instructions. Reactions were conducted in triplicates, and relative gene expression levels were determined using the 2^−ΔΔCt^ method, with GAPDH as the internal reference gene. The primers used for qRT-PCR amplification are detailed in the **Supplementary Material**.

### 2.9 Statistical Analysis

Western blot analysis was repeated three times using individually cultured cells. Other *in vitro* experiments were performed once in triplicate. For *in vivo* experiments, the total number of mice used is indicated in the figure legends. Data are expressed as means ± standard deviations (SDs), and normality was assessed using the Shapiro-Wilk test, and the data were confirmed to follow a normal distribution. Then statistical analysis was performed using one-way ANOVA and Tukey’s honestly using GraphPad Prism 9 (GraphPad Software Inc, San Diego, CA, USA). p-values were expressed as follows: * p < 0.05, ** p < 0.01, and *** p < 0.001.

## 3 Results

### 3.1 C-di-AMP inhibits bone destruction in ligation-induced periodontitis

During the animal experiments, we collected tissue samples from the alveolar bone at 1, 2, and 3 weeks, respectively. The distance from the cemen-to-enamel junction (CEJ) to the alveolar bone crest (ABC) was measured, and the difference between the measured value and the corresponding value on the untreated control side (Con side) was calculated as the ΔCEJ-ABC, serving as an indicator of the severity of alveolar bone resorption. At the 3-week mark, ligatures were observed present between the maxillary molars of the mice. The sampling image reveals the intact separation of the murine alveolar bone with gingival tissue (**Figures 1A, B**).

Micro-CT analysis with images and CEJ-ABC revealed significant alveolar bone resorption in all periodontitis side (PD side) of experimental groups, with an increasing severity of bone loss over time. The results indicate that there were no significant intergroup differences or temporal changes in the CEJ-ABC distance on the control (Con) side across all groups (**Figures 1C, D**). Further analysis with ΔCEJ-ABC demonstrated that prophylactic administration of c-di-AMP in mice provided protection against periodontitis compared to the PBS group (**Figure 1E**). Notably, it significantly reduced alveolar bone resorption and prevented the loss of bone density (**Appendix Figure 1**). Among the different concentrations tested, the effect of c-di-AMP was particularly pronounced in the 10 μmol/L group.

The results of HE staining also demonstrated significant increases in alveolar bone resorption and a notable decrease in the height of the alveolar crest in the ligature-induced PD side of PBS group compared to the Con side. Additionally, epithelial hyperplasia was evident in the gingival tissue of the PD side. In contrast, the 10 μmol/L group exhibited relatively milder alveolar bone resorption in the distal furcation region of the maxillary first molar, with less reduction in the alveolar crest height and no apparent epithelial hyperplasia. The 100 μmol/L group also showed improvement in alveolar bone resorption, although the effect was less pronounced compared to the 10 μmol/L group (**Figure 1C**).

### 3.2 C-di-AMP promotes the expression and phosphorylation of Akt in human gingival epithelial cells

**Figure 2:**
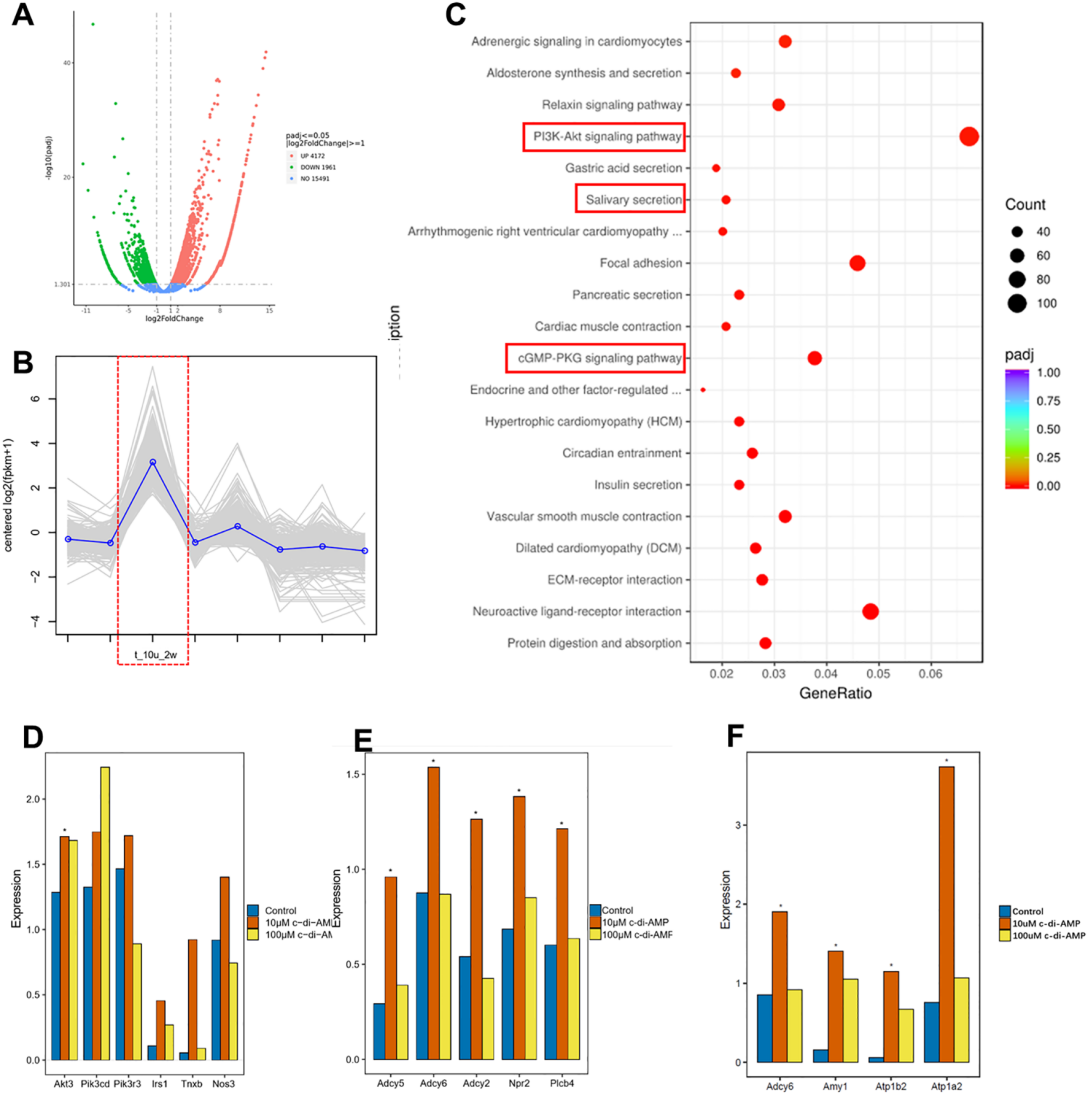
C-di-AMP promotes the expression of the PI3K/Akt signaling pathway in murine gingival tissue. **(A)** Differential gene expression analysis. **(B)** Hierarchical cluster analysis. **(C)** KEGG enrichment analysis: top 20 enriched up-regulated pathways. The expression of key component genes of **(D)** the PI3K-Akt signaling pathway, **(E)** cGMP/PKG signaling pathway, **(F)** Salivary secretion pathway. *p < 0.05.

**Figure 3:**
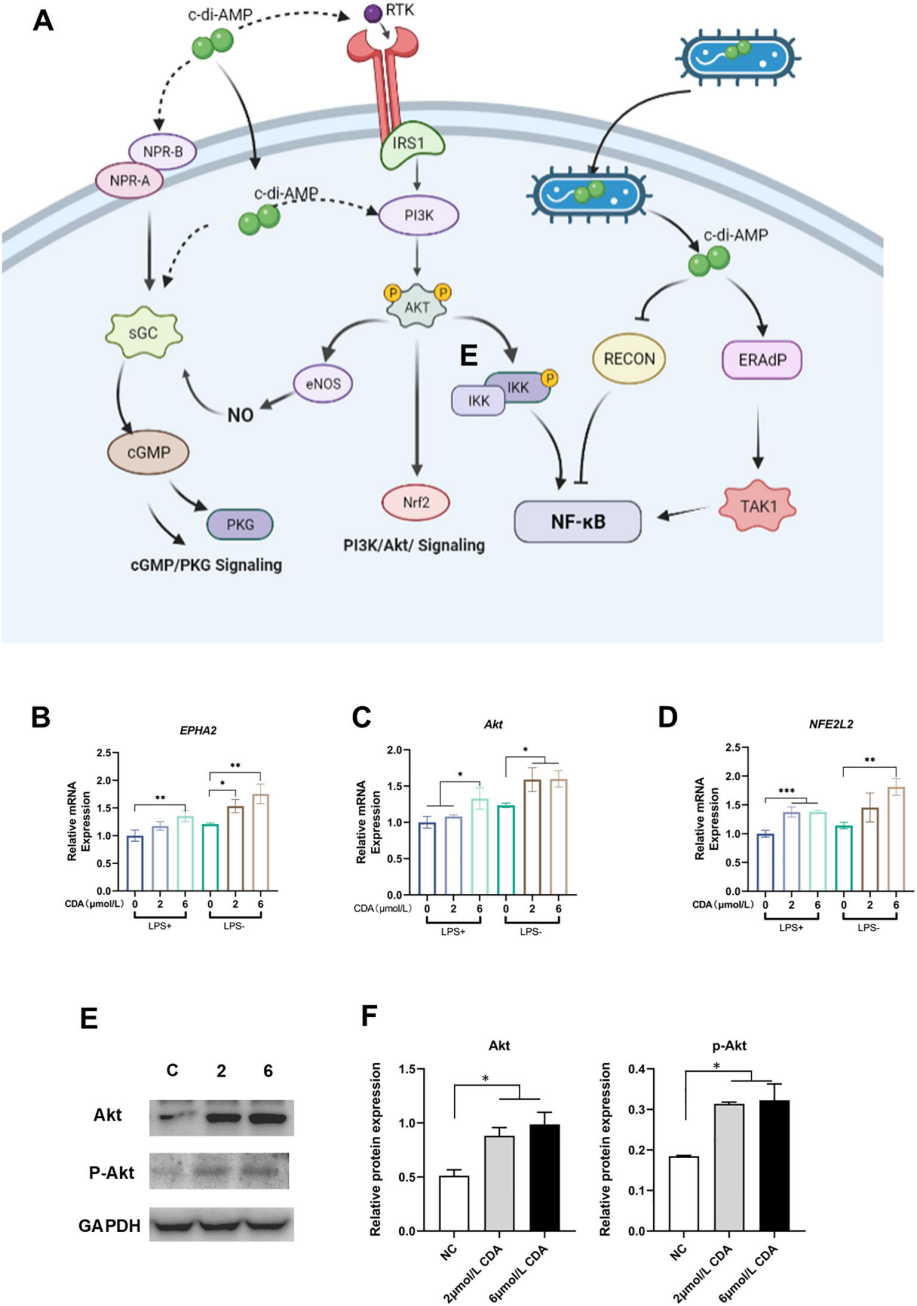
C-di-AMP promotes the expression and phosphorylation of Akt in Human gingival epithelial cells. **(A)** The schematic diagram illustrates the cellular mechanism of c-di-AMP action on host cells. It shows a host cell with various receptors on its surface. c-di-AMP, as a bacterial second messenger, can enter the host cell either through the cell membrane or via receptor-mediated endocytosis. Inside the cell, c-di-AMP interacts with intracellular receptors, including STING (not showed in this diagram), PI3K/Akt signaling pathway and other signaling proteins. C-di-AMP can activate the PI3K/Akt signaling pathway, which regulates various cellular processes, including cell survival and proliferation. Activated Akt further activates nuclear factor erythroid 2-related factor (Nrf2), promoting the expression of genes involved in antioxidative stress and anti-inflammatory responses. The diagram also indicates that c-di-AMP may form a positive feedback loop between the PI3K/Akt pathway and the cGMP/PKG pathway. This feedback loop involves the activation of nitric oxide synthase (NOS), leading to increased production of nitric oxide (NO). The elevated NO activates the NO-cGMP-PKG pathway, which is crucial for regulating bone homeostasis. The relative expression levels of key component genes of PI3K/Akt pathway, **(B)** EPHA2, **(C)** Akt, and **(D)** NFE2L2. **(E)** Western blot analysis for Akt, p-Akt expression. **(F)** The relative protein expression levels of Akt, p-Akt. *p < 0.05, **p < 0.01, ***p < 0.001.

To investigate the effects of c-di-AMP treatment on gene expression in periodontal tissue, we collected gingival tissue samples from the local area of periodontitis for transcriptome sequencing analysis. Our primary objective was to explore the changes in the transcriptomic profile of the gingival tissue after treatment with c-di-AMP (CDA), with a specific focus on inflammation-related signaling pathways. To identify differentially expressed genes between the 10 μmol/L CDA group, which exhibited a greater reduction in alveolar bone resorption, with the PBS group, we conducted differential gene expression analysis (**Figure 2A**). We performed hierarchical clustering of the differentially expressed genes based on their expression patterns and focused on analyzing the cluster that covered the highest number of genes and displayed the significant trend. The results revealed that this cluster consisted of 353 genes, with significantly upregulated gene expression in the 10 μmol/L CDA 2-week group, consistent with the results from the animal experiment (**Figure 2B**). Through KEGG enrichment analysis, we identified the top 20 enriched pathways. Among them, the PI3K/Akt signaling pathway and cGMP/PKG signaling pathway were found to be significantly upregulated in the 10 μmol/L CDA group (**Figure 2C**). Subsequently, we conducted an analysis of the transcript levels of key components genes within the relevant signaling pathways (**Figures 2D–F**). Specifically, the 10 μmol/L CDA group exhibited significant upregulation of key component genes (Irs1, Akt3, PIK3r3, Tnxb, Nos3) in the PI3K-Akt signaling pathway in gingival tissues under periodontal inflammation conditions (**Figure 2D**). Among them, the Akt kinase encoded by the Akt3 gene is a crucial signal regulator in the PI3K/Akt signaling pathway, which is associated with the activation of the immune response against infections.

c-di-AMP, as a bacterial second messenger, can enter the host cell either through the cell membrane or via receptor-mediated endocytosis (Devaux et al., 2018). As show in diagram, c-di-AMP can bind to the membrane protein receptor tyrosine kinase (RTK) through either extracellular or intracellular mechanisms, triggering the activation of the PI3K/Akt signaling pathway. This activation leads to increased expression and phosphorylation of Akt kinase, the key protein in PI3K/Akt. Phosphorylated Akt (p-Akt) further activates nuclear factor erythroid 2 related factor (Nrf2). The activation of Nrf2 is associated with the expression of genes involved in antioxidant stress response and anti-inflammatory processes (**Figure 3A**). In recent years, Nrf2 has also been found to be related to the activation of innate immunity and the expression of immune-related genes.

To validate our findings, we conducted experiments using human gingival epithelial cells, which are most relevant to the microenvironment of periodontitis. After treating the cells with c-di-AMP, we observed significant upregulation of key genes in the PI3K/Akt pathway, including EPHA2, Akt, and NFE2L2 (**Figures 3B–D**). Western blot analysis confirmed a significant increase in the protein levels of Akt and phosphorylated Akt, providing further evidence that the PI3K/Akt pathway can be activated by c-di-AMP and plays a role in the local environment of periodontitis (**Figure 3E, F**).

### 3.3 C-di-AMP suppresses the LPS-induced inflammatory response of gingival epithelial cells through PI3K/Akt pathway

**Figure 4:**
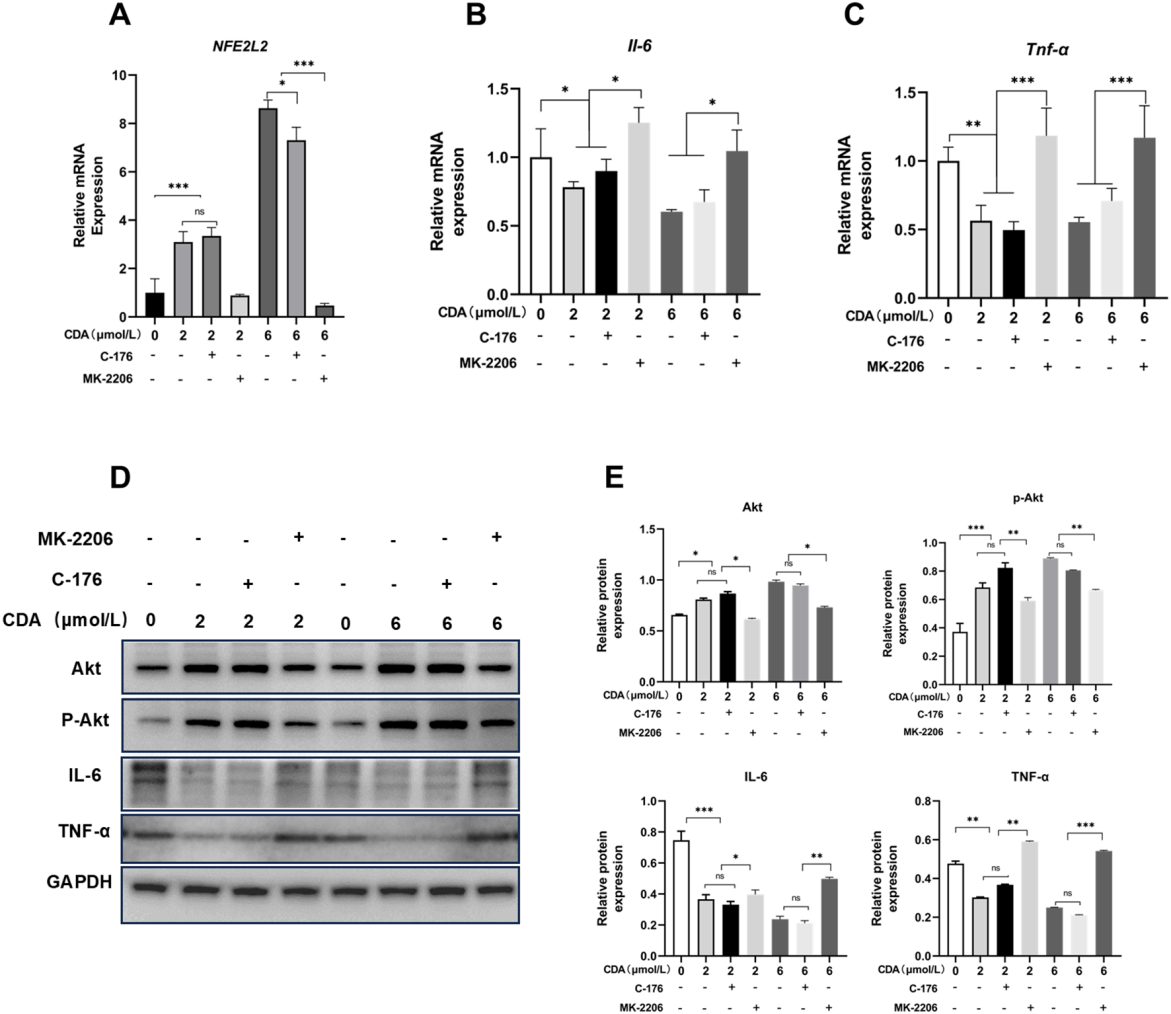
C-di-AMP suppresses the LPS-induced inflammatory response of gingival epithelial cells through PI3K/Akt pathway. HGEC were exposed to c-di-AMP along with the AKT inhibitor MK-2206 and STING inhibitor C176, and LPS was pre-used to induce inflammation. The relative mRNA expression level of **(A)** Nuclear factor erythroid 2-related factor 2 (Nrf2, NFE2L2), **(B)** interleukin (IL)–6, **(C)** tumor necrosis factor α (TNF-α). Western blot analysis for Akt, p-Akt, IL-6 and TNF-α expression **(D, E)**. *p < 0.05, **p < 0.01, ***p < 0.001.

We have demonstrated the ability of c-di-AMP to activate Akt in human gingival epithelial cell (HGEC). Considering the STING pathway has been previously identified as one of the main mechanisms through which c-di-AMP exerts its effects, we hypothesized that the activation of Akt may be associated with the interaction between the STING pathway and Akt in this experimental setting. To further elucidate the anti-inflammatory capacity of c-di-AMP in cellular experiments, we conducted follow-up experiments where HGEC were exposed to c-di-AMP along with the Akt inhibitor MK-2206 and STING inhibitor C176, with LPS pre-treatment to induce an inflammatory environment. This approach aims to confirm the pathways underlying c-di-AMP’s anti-inflammatory effects while evaluating its mechanistic role in cellular models. Real-time quantitative PCR was used to detective the expression of Nrf2 and inflammatory cytokines including IL-6 and TNF-α. Upon investigation, we have observed that in the presence of an inflammatory environment, c-di-AMP can consistently activate Nrf2, thereby inhibiting the production of IL-6 and TNF-α, ultimately exerting an anti-inflammatory effect. Interestingly, this effect seems to be independent of the inhibition of the STING pathway. However, when Akt is suppressed, the levels of Nrf2 significantly decrease, while the levels of IL-6 and TNF-α might even slightly increase compared to pre-treatment with c-di-AMP (**Figures 4A-C**). Our hypothesis was further confirmed through Western blot experiments, which indicated that c-di-AMP suppresses the inflammatory response of gingival epithelial cells by activating Akt. Notably, when the Akt pathway is inhibited, both Akt and p-Akt levels significantly decrease, while the protein levels of the inflammatory factors IL-6 and TNF-α increase (**Figures 4D, E**).

### 3.4 c-di-AMP exhibits no significant influence on periodontal microbiota

**Figure 5:**
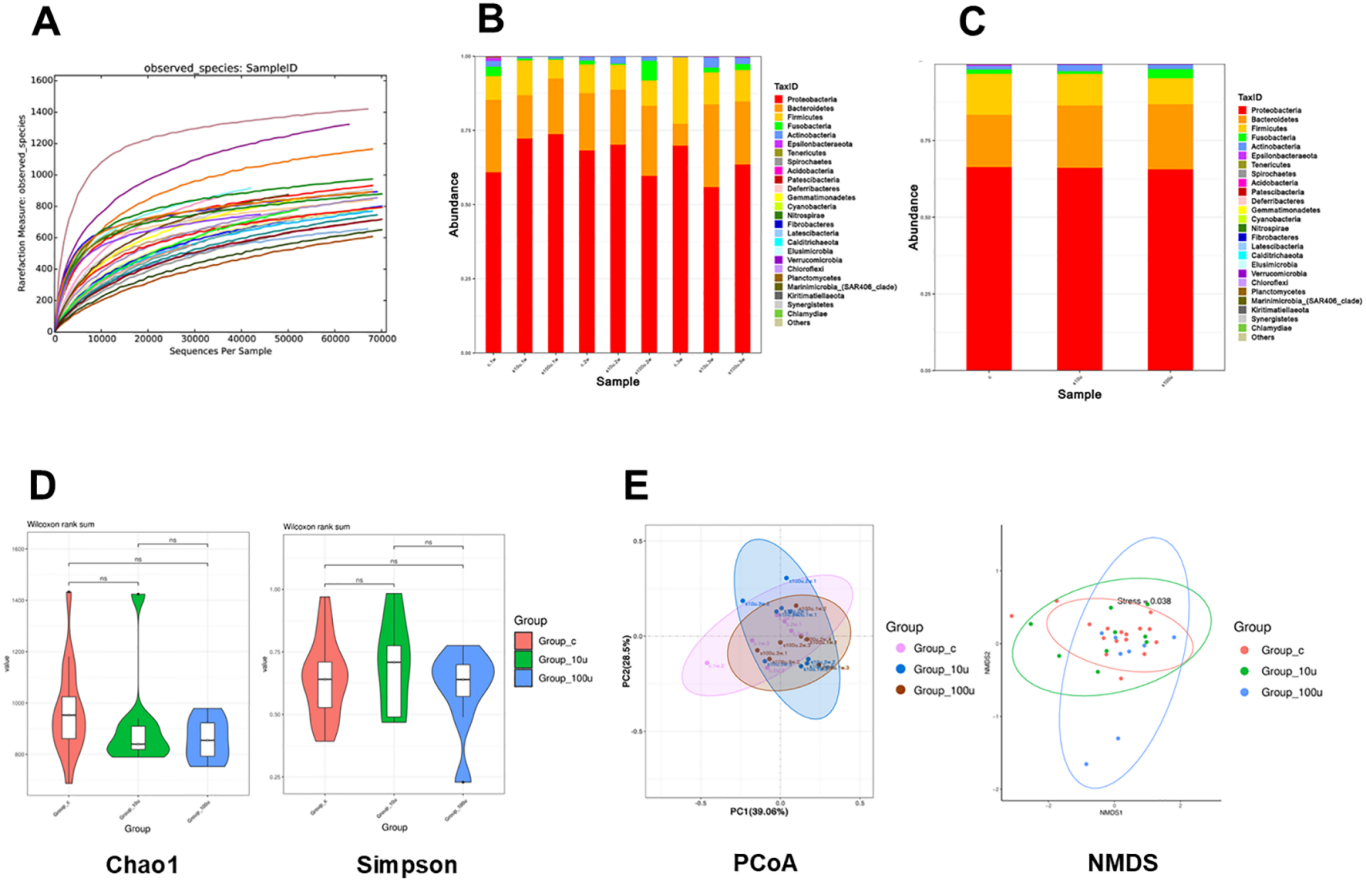
Prophylactic use of c-di-AMP exhibits no significant influence on oral microbiota in periodontitis. **(A)** Diversity index dilution curve indicate sufficient sequencing depth. Relative abundance of the oral microbiota at the **(B)** phylum level and **(C)** genus level. **(D)** The α diversity using the Chao1 index and Simpson index. **(E)** The β diversity using PCoA analysis and NMDS analysis based on Bray–Curtis distance of oral microbiota among 3 groups.

The 16S rRNA gene sequencing demonstrated that there were no significant changes in the composition of the oral microbiota in the mice with periodontitis before and after treatment with c-di-AMP (**Figures 5A-C**). At the phylum level, the distribution of relative abundances (≥1%) in the oral microbiota of all samples showed the following order: Proteobacteria (64.3%), Bacteroidetes (21.4%), Firmicutes (12.1%), Fusobacteria (1.5%), and Actinobacteria (1.4%) (**Figure 5C**). Estimates of richness (observed species, Chao1), richness/evenness (Simpson) were used to assess α-diversity and indicated no significant changes in microbial richness and evenness in the local periodontal microbiota (**Figure 5D**). Beta diversity analysis, conducted using principal-coordinate analysis and Nonmetric Multidimensional scaling (NMDS), consistently revealed no significant differences in microbial composition among the three groups (**Figure 5E**).

To determine the impact of c-di-AMP on the abundance of periodontal pathogens in the local environment of periodontitis, we analyzed the changes in abundance at the genus level. The results revealed slight reductions in the abundance of *Porphyromonas, Prevotella_9, Fusobacterium, Neisseria, and Actinomyces*, along with a slight increase in the abundance of *Streptococcus* in the local periodontal tissues. However, these differences were not statistically significant, suggesting that c-di-AMP had no significant impact on the abundance of periodontal pathogens in the local environment of periodontitis (**Appendix Figure 3**).

## 4 Discussion and conclusion

The disruption of the balance between dysbiotic oral microbial communities and host immune defense is a critical mechanism underlying the development of periodontitis (Kinane et al., 2017). C-di-AMP, a bacterial second messenger present in oral bacteria and dental biofilms (Peng et al., 2017, 2016), not only regulates various physiological functions within oral bacteria but also activates host immune responses through multiple pathways, promoting the defense against infections (Cheng et al., 2022). In this study, we conducted a preliminary investigation into the effects and mechanisms of c-di-AMP in periodontitis.

Given the irreversible nature of alveolar bone resorption caused by periodontal inflammation, controlling the progression of alveolar bone resorption is of utmost importance in the clinical management of periodontitis (Zhou and Graves, 2022). The ligature-induced mice model enables the induction of host inflammatory responses and alveolar bone resorption in a specific location (ligature site) within a short period of time (Marchesan et al., 2018). With the assistance of tissue sectioning and Micro CT, both the inflammatory response and alveolar bone resorption can be observed and quantitatively measured (Tamura et al., 2021). In this study, we discovered that topically applying c-di-AMP in periodontal tissues inhibited alveolar bone resorption in mice periodontitis. Moreover, c-di-AMP reduced attachment loss caused by the reduction of the alveolar crest caused by local periodontal inflammation.

Through animal and cellular experiments, we further discovered that in periodontitis, c-di-AMP tends to regulate inflammation through the PI3K/Akt/Nrf2 pathway, which promotes host immune defense and bone homeostasis, ultimately inhibiting periodontitis progression and alveolar bone resorption. Interestingly, the STING pathway was not significantly activated, suggesting local receptors in the periodontal area may be involved (Elmanfi et al., 2021). Previous studies also show c-di-AMP can activate non-STING pathways in specific contexts (Aryal et al., 2020; Sooreshjani et al., 2018). The PI3K/Akt pathway plays an important role in cell survival and responses to extracellular stimuli like cytokines. Once activated, PI3K induces Akt phosphorylation, which then regulates cellular phenotypes including proliferation and apoptosis (Glaviano et al., 2023). The PI3K/Akt pathway is involved in immune responses to infections. Akt activates Nrf2, which expresses antioxidant and anti-inflammatory genes. Nrf2 protects against tissue destruction in periodontitis by regulating reactive oxygen species (Li et al., 2023). Akt also activates NF-κB, a transcription factor that regulates immune recognition receptors, cytokines, and other immune mediators. Previous studies show c-di-AMP activates NF-κB to induce innate immunity (McFarland et al., 2017). We found c-di-AMP upregulates NF-κB genes in gingival cells, likely promoting local immune responses in periodontitis.

The cGMP-PKG pathway crucially regulates bone homeostasis, with its activation promoting osteogenic differentiation while simultaneously inhibiting osteoclast activity. In our study, we observed upregulation of cGMP-PKG signaling in response to c-di-AMP, suggesting its potential role in modulating bone metabolism. The NO–cGMP–PKG axis is particularly important in skeletal remodeling, as nitric oxide (NO) signaling leads to cGMP production and PKG activation, which mediate the anabolic effects of mechanical stimuli and estrogen on bone formation. Furthermore, PKG activation has been shown to restore osteoblast function and promote bone formation even under inflammatory conditions (Kim et al., 2021). Based on these findings, we hypothesize that interactions between PI3K/Akt and cGMP/PKG signaling form a positive feedback loop involving NO production, which could contribute to bone homeostasis and periodontal disease modulation. Specifically, PI3K/Akt activation enhances NO synthase (NOS) activity and increases NO levels, which in turn activates cGMP-PKG signaling. This activation further promotes osteoblast differentiation and bone formation, creating a self-reinforcing regulatory loop. We propose that this mechanism may explain how c-di-AMP inhibits osteoclast activity, stimulates osteoblast differentiation, and ultimately reduces alveolar bone resorption (Zhai et al., 2014). Interestingly, a similar cGMP-PKG/PI3K-Akt positive feedback loop has been reported in vascular endothelial cells. In studies on pulmonary vasodilation, PKG activation was found to regulate Akt phosphorylation through protein phosphatase 1 (PP1), leading to increased cGMP levels via PDE5 inhibition (Liu et al., 2014). In conclusion, our findings indicate that c-di-AMP may leverage the cGMP-PKG/PI3K-Akt feedback loop to regulate osteoclast and osteoblast activity, thereby contributing to alveolar bone preservation. Further research is required to experimentally validate this feedback mechanism in periodontal disease models and determine its potential therapeutic implications.

In addition, we found that c-di-AMP could regulate the expression of inflammatory factors, which provide evidence that c-di-AMP can modulate host immunity and bone homeostasis to mitigate periodontal tissue damage. Oral microbial communities and host immune defense are two key factors in the development of periodontitis. We initially hypothesized that c-di-AMP may alter the local microbiota composition in mice with periodontitis, promoting microbial ecological improvement and inhibiting disease progression. However, 16S rRNA gene sequencing showed no significant changes in the local periodontal microbiota of mice before and after c-di-AMP treatment. We observed a slight decrease in putative periodontal pathogens after c-di-AMP treatment, although these differences were statistically insignificant.

In our previous investigation exploring the relationship between c-di-AMP and atherosclerosis, we discovered that c-di-AMP could prevent changes in the oral microbiota composition induced by *P. gingivalis* infection in ApoE-/- mice. This preliminary finding suggested a potential role of c-di-AMP in maintaining periodontal homeostasis in mice (Wu et al., 2023). In this study, without the active intervention of adding *P. gingivalis*, we did not observe significant changes in the local oral microbial ecology of mice before and after the use of c-di-AMP. It may provide additional evidence that c-di-AMP may help maintain the mice’s microbiota in its initial state. The alterations in the microbiota appeared to play a secondary role in the establishment of experimental periodontitis in this study. The specific mechanism by which c-di-AMP alters microbial composition remains unclear. Further research is needed to explore the interaction between c-di-AMP and microorganisms, as well as the regulatory mechanisms of c-di-AMP on the microbial community structure. The lack of significant microbial compositional changes indicates that c-di-AMP mediates its protective effects against periodontitis primarily through direct modulation of host immunity rather than the oral microbiota.

While our study provides valuable insights into the protective effects of c-di-AMP against periodontitis through activation of the PI3K/Akt/Nrf2 pathway, there are several limitations that should be acknowledged. First, the ligature-induced model, though commonly used, may not fully recapitulate the chronic complexity and host-microbiome dynamics of human periodontitis (Guo et al., 2021). Second, while transcriptomic analysis and *in vitro* experiments suggest that c-di-AMP exerts its anti-inflammatory effects through PI3K/Akt/Nrf2 activation, direct *in vivo* confirmation of this mechanism is lacking. Future studies could employ genetic knockouts or specific inhibitors of the PI3K/Akt pathway in mice to validate the causality of this mechanism. Lastly, the observed upregulation of cGMP-PKG signaling requires further investigation to clarify its precise interaction with PI3K/Akt, given its known but incompletely defined role in osteogenic differentiation and bone homeostasis.

Despite these limitations, our findings offer valuable insight into how c-di-AMP modulates immune defense and bone stability by activating PI3K/Akt, Nrf2, and potentially cGMP-PKG. These results open new avenues for developing targeted periodontal therapies that go beyond conventional mechanical debridement. Future work employing advanced animal models, genetic manipulations, and extended treatment durations could validate and refine c-di-AMP based strategies, potentially translating into clinical interventions that more effectively preserve periodontal health.

## Data Availability

The datasets presented in this study can be found in online repositories. The names of the repository/repositories and accession number(s) can be found below: www.ncbi.nlm.nih.gov/bioproject/?term=1126755.
