# Supplementary material for: Cyclic di-AMP alleviates periodontitis by activating PI3K/Akt/Nrf2 pathways
Source: Front Cell Infect Microbiol. 2025 Mar 14;15:1560155. doi: 10.3389/fcimb.2025.1560155 (PMC11949975; doi:10.3389/fcimb.2025.1560155)
Supplement: Supplementary file 1 [file DataSheet1.docx]

Supplementary Material

Cyclic di-AMP alleviates periodontitis by activating PI3K/Akt/NrF2 pathways

Kaihua Luo^1, 2^ , Qinrui Wu^1, 2^, Zhengyi Li^1^, Yajie Wu^1, 2^, Zhifei Su^1, 2^, Fangjie Zhou^1, 2^, Qinyang Li^1, 2^, Biao Ren^1^, Yuqing Li^1^, Jiyao Li^1, 2*^, Xian Peng^1*^

^1^State Key Laboratory of Oral Diseases & National Center for Stomatology & National Clinical Research Center for Oral Diseases, West China Hospital of Stomatology, Sichuan University, Chengdu 610041, Sichuan, China

^2^Department of Cariology and Endodontics, West China Hospital of Stomatology, Sichuan University, Chengdu 610041, Sichuan, China

*** Correspondence:**Jiyao Li, jiyaoliscu@163.com; Xian Peng, pengx@scu.edu.cn;

# Materials and methods:

## Reagents

The c-di-AMP (purity ≥ 98%) was obtained from Sigma-Aldrich technology, Co. (Shanghai, China) and diluted in PBS for in vivo and in vitro experiments. LPS from *Porphyromonas gingivalis* was obtained from Sigma-Aldrich technology, Co. (Shanghai, China). The Akt inhibitor MK-2206 and the STING inhibitor C176 were purchased from APExBIO (USA). Stock solutions of MK-2206 (10 mM) and C176 (10 mM) were prepared in dimethyl sulfoxide (DMSO) and further diluted in PBS to achieve final working concentrations of 1 µM for MK-2206 and 1 µM for C176 in experimental conditions.

## Animal Experiments

Six-week-old male healthy C57BL/6 mice were purchased from Chengdu Dossy Experimental Animals Co., Ltd. (Chengdu, China) and fed in a temperature-controlled room, with a 12 h light-dark cycles. The study was conducted according to the guidelines of ARRIVE and the experimental protocols were approved by the Animal Care and Use Committee of West China Hospital of Stomatology Hospital, Sichuan University, Chengdu China (WCHSIRB-D-2020-031).

After a 2-week acclimatization period, mice were randomly divided into three groups (n = 9 per group, with 3 mice assessed at each designated time point): control, 10 μM c-di-AMP and 100 μM c-di-AMP intervention. The concentrations of 10 μM and 100 μM were selected based on preliminary pilot studies and our previous work (Wu et al., 2023). Mice were first anesthetized with isoflurane (5% for induction and 2% for maintenance) delivered via an anesthesia chamber, a method widely used in rodent studies due to its rapid onset and precise control of anesthesia depth. A ligation-induced periodontitis model was then established on the left side, with the control group receiving 0.9% NaCl solution and the intervention groups receiving either 10 μM or 100 μM c-di-AMP solution. After model establishment, 25 μL of the respective solution was locally injected at six sites around the first maxillary molar. Ligatures were checked every two days. The experiments were started with mice at 8-week of age after 1-week of adaptive feeding and terminated at the 11th week (Fig. 1A). The study was conducted in two independent experimental rounds to ensure the reproducibility of the results.

All mice were sacrificed humanely using established protocols to ensure they were anesthetized and unconscious before sample collection. Euthanasia was performed using an overdose of pentobarbital sodium. Mice were administered an intraperitoneal injection of pentobarbital sodium at a dose of 150 mg/kg body weight. This dosage ensured rapid induction of deep anesthesia followed by euthanasia. Death was confirmed by the absence of heartbeat and respiration, and cervical dislocation was performed as a secondary method to ensure death.

Alveolar bones and gingival tissue were harvested from the mice, postfixed with 4% paraformaldehyde overnight, and stored in 70% ethanol at 4 °C for long-term storage. Silk samples were carefully collected before euthanasia in sterile Eppendorf tubes and kept at -80℃ for future analysis. Alveolar bone tissue was collected from all mice for μCT analysis (Miyajima et al. 2014; Morelli et al. 2018; Rekhi et al. 2021). And only 2-week alveolar bone with gingival tissue were collected for H&E staining (Leite et al. 2017; Marchesan et al. 2018).

## Micro-Computed Tomography Analysis

The microarchitectural properties of alveolar bone tissues were imported with µCT system. Parameter settings were as follows: the scanning was performed at 70 kV and 200 μA with 300 ms integration time at a voxel resolution of 10 μm. The images were reconstructed using mimics (version 21.0, Materialise N.V., Leuven, Belgium). The results of BV/TV, BMD, and CEJ-ABC were obtained within the region of interest, which surrounds the second molar. CEJ-ABC was determined by measuring the average distance between the alveolar bone crest (ABC) and the cementoenamel junction (CEJ) in the sagittal images. And ΔCEJ-ABC refers to the difference between CEJ-ABC on the left side and CEJ-ABC on the right side. Trabecular bone volume fraction (Bone volume/Total volume, BV/TV) was used to analyze the degree of trabecular bone resorption.

## Hematoxylin and Eosin Staining

To evaluate the severity of the periodontitis condition and the height of the alveolar crest, Hematoxylin and Eosin (H&E) Staining was performed. Maxillary bone samples were fixed in 4% paraformaldehyde at 4°C for 24 h, decalcified in 10% EDTA (pH 7.4) for 4 weeks, embedded in paraffin, and sectioned into 10-μm sagittal slices. Sections were deparaffinized, rehydrated, stained with hematoxylin (5 min) and eosin (30 sec), dehydrated, cleared in xylene, and mounted. Alveolar bone resorption was assessed by identifying the alveolar bone crest (ABC) position on H&E-stained sections, with a black dashed line marking its approximate height.

## 16S rRNA Sequencing and Analysis

The microbiomes of silk ligatures were analyzed using 16S ribosomal RNA (rRNA) sequencing. Total genomic DNA was extracted using the QIAamp DNA Mini Kit (QIAGEN) following the manufacturer’s instructions. DNA concentration and purity were assessed using a NanoDrop 2000 (Thermo Fisher Scientific), ensuring an average yield of ≥20 ng/µL, and integrity was verified by agarose gel electrophoresis. The genome DNA was used as template for PCR amplification with the barcoded primers and Tks Gflex DNA Polymerase (Takara). For bacterial diversity analysis, V3-V4 variable regions of 16S rRNA genes was amplified with universal primers 343F and 798R performed by oebiotech Technology Co., Ltd, Shanghai, China.

The microbiomes of silk ligatures were analyzed using 16S ribosomal RNA (rRNA) sequencing. Total genomic DNA was extracted using the QIAamp DNA Mini Kit (QIAGEN) following the manufacturer’s instructions. DNA concentration and purity were assessed using a NanoDrop spectrophotometer (Thermo Fisher Scientific), ensuring an average yield of ≥20 ng/µL, and integrity was verified by agarose gel electrophoresis. Purified DNA was used as a template for PCR amplification of the V3-V4 hypervariable regions of the 16S rRNA gene using barcoded universal primers 343F and 798R and Tks Gflex DNA Polymerase (Takara, Japan).

## 16S rRNA Sequencing and Analysis

Total RNA was extracted from gingival tissue and human gingival epithelial cells using the TRIzol reagent (Invitrogen, USA) following the manufacturer’s protocol. RNA purity and concentration were assessed using a NanoDrop spectrophotometer (Thermo Fisher Scientific, USA), and RNA integrity was evaluated with an Agilent 2100 Bioanalyzer (Agilent Technologies, USA) to ensure an RNA integrity number (RIN) ≥ 7.0. For library construction, mRNA was enriched from total RNA using oligo (dT) magnetic beads, followed by fragmentation, cDNA synthesis, and adapter ligation using the NEBNext Ultra RNA Library Prep Kit (New England Biolabs, USA). The purified libraries were PCR-amplified, and final sequencing libraries were quantified using a Qubit Fluorometer (Thermo Fisher Scientific, USA) and validated with an Agilent 2100 Bioanalyzer.

## Cell Cultures

Human gingival epithelial cells (HGECs, 3rd passage) were obtained from Warner Biotechnology Co., Ltd. HGECs were cultured and seeded at a density of 1 × 10^5^/mL in a growth medium (DMEM) in 6-well plates overnight. Different doses of c-di-AMP (0, 2, 6 μM) were added to the HGECs, and the samples were incubated for 24h.

## qRT-PCR

To validate gene expression, qRT-PCR was performed using the SYBR Green PCR Master Mix (Applied Biosystems, USA) on a QuantStudio 5 Real-Time PCR System (Thermo Fisher Scientific, USA). And cDNA was synthesized using RevertAid First Strand cDNA Synthesis Kit (Thermo Fisher Scientific, USA) following the manufacturer’s instructions. Reactions were conducted in triplicates, and relative gene expression levels were determined using the 2^−ΔΔCt^ method, with GAPDH as the internal reference gene. The primers used for qRT-PCR amplification see Appendix table 1.

## Statistical Analysis

Western blot analysis was repeated three times using individually cultured cells. Other in vitro experiments were performed once in triplicate. For in vivo experiments, the total number of mice used is indicated in the figure legends. Data are expressed as means ± standard deviations (SDs), and normality was assessed using the Shapiro-Wilk test, and the data were confirmed to follow a normal distribution. Then statistical analysis was performed using one-way ANOVA and Tukey’s honestly using GraphPad Prism 9 (GraphPad Software Inc, San Diego, CA, USA). p-values were expressed as follows: * p < 0.05, ** p < 0.01, and *** p < 0.001.

# Supplementary Figures and Tables


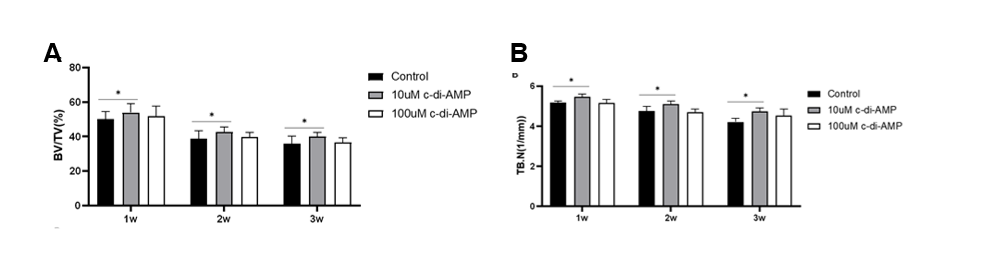


**Appendix Figure 1. C-di-AMP inhibits bone destruction in periodontitis induced by ligature placement in mice.** (A) bone volume fraction (bone volume/total volume). (B) bone trabecular number (Tb. N).


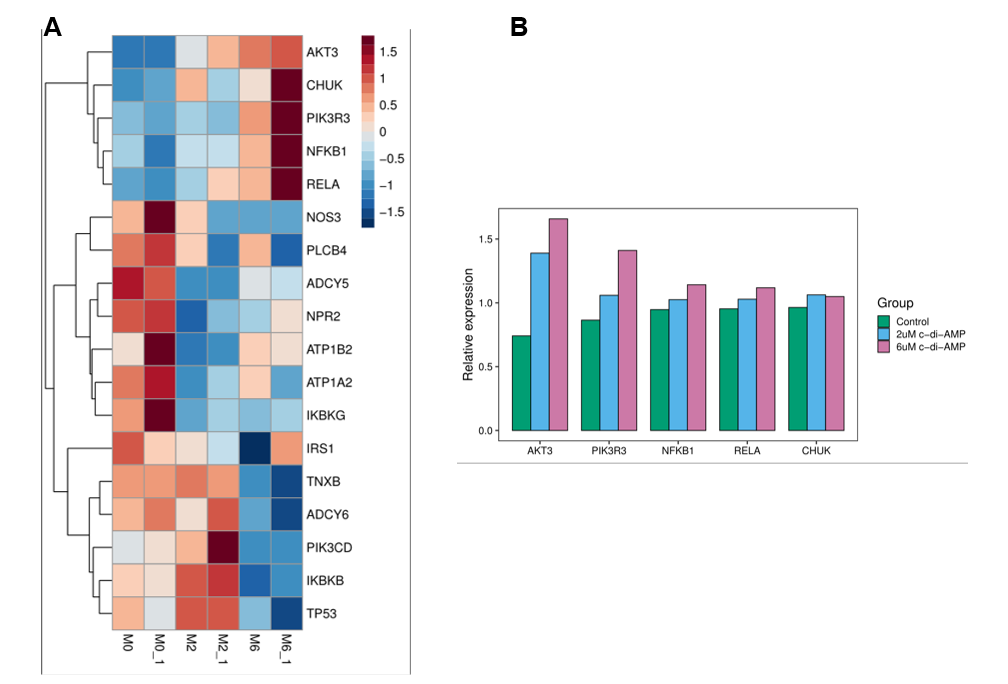


**Appendix Figure 2. C-di-AMP promotes the expression of the PI3K/Akt signaling pathway in human gingival epithelial cells.** (A) Heatmap of differential gene expression. (B) The expression of top 5 enriched up-regulated genes.


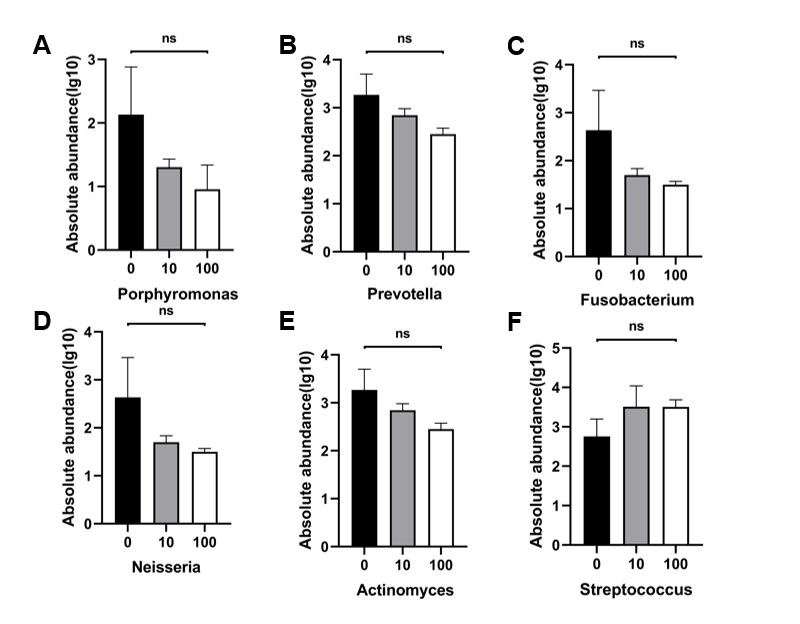


**Appendix Figure 3. C-di-AMP exhibits no significant influence on oral microbiota in periodontitis.** The absolute abundance of (A) Porphyromonas, (B) Prevotella_9, (C) Fusobacterium, (D) Neisseria, (E) Actinomyces, (F) Streptococcus.

**Appendix table 1. Primers of RT-qPCR**

| Primers | Forward (5’ to 3’) | Reverse (3’ to 5’) |
| --- | --- | --- |
| GAPDH | AATCCCATCACCATCTTCCAG | AAATGAGCCCCAGCCTTC |
| IL-6 | ACTCACCTCTTCAGAACGAATTG | CCATCTTTGGAAGGTTCAGGTTG |
| TNF-α | CCTCTCTCTAATCAGCCCTCTG | GAGGACCTGGGAGTAGATGAG |
| EPHA2 | TGGCTCACACACCCGTATG | GTCGCCAGACATCACGTTG |
| AKT | AGCGACGTGGCTATTGTGAAG | GCCATCATTCTTGAGGAGGAAGT |
| NFE2L2 | TCAGCGACGGAAAGAGTATGA | CCACTGGTTTCTGACTGGATGT |
| 16srRNA | TACGGRAGGCAGCAG | AGGGTATCTAATCCT |

Leite FRM, Peres KG, Do LG, Demarco FF, Peres MAA. 2017. Prediction of periodontitis occurrence: Influence of classification and sociodemographic and general health information. Journal of periodontology. 88(8):731-743.

Marchesan J, Girnary MS, Jing L, Miao MZ, Zhang S, Sun L, Morelli T, Schoenfisch MH, Inohara N, Offenbacher S et al. 2018. An experimental murine model to study periodontitis. Nature protocols. 13(10):2247-2267.

Miyajima S, Naruse K, Kobayashi Y, Nakamura N, Nishikawa T, Adachi K, Suzuki Y, Kikuchi T, Mitani A, Mizutani M et al. 2014. Periodontitis-activated monocytes/macrophages cause aortic inflammation. Sci Rep. 4:5171.

Morelli T, Moss KL, Preisser JS, Beck JD, Divaris K, Wu D, Offenbacher S. 2018. Periodontal profile classes predict periodontal disease progression and tooth loss. Journal of periodontology. 89(2):148-156.

Rekhi UR, Catunda RQ, Alexiou M, Sharma M, Fong A, Febbraio M. 2021. Impact of a cd36 inhibitor on porphyromonas gingivalis mediated atherosclerosis. Arch Oral Biol. 126:105129.
